# NOURISH-US: a mixed-methods, randomized crossover study of a program designed to reduce the financial burden of food allergy

**DOI:** 10.1186/s13223-025-00983-2

**Published:** 2025-08-21

**Authors:** Michael A. Golding, Sarah Baldwin, Brandon Kim, Zoe Harbottle, Manvir Bhamra, Dylan S. Mackay, Moshe Ben-Shoshan, Jennifer D. Gerdts, Elissa M. Abrams, Sara J. Penner, Jo-Anne St-Vincent, Jennifer L. P. Protudjer

**Affiliations:** 1 The Children’s Hospital Research Institute of Manitoba, Winnipeg, MB Canada; 2 Department of Pediatrics and Child Health, University of Manitoba, Winnipeg, MB Canada; 3 Rady Faculty of Health Sciences, Max Rady College of Medicine, University of Manitoba, Winnipeg, MB Canada; 4 Department of Food and Human Nutritional Sciences, University of Manitoba, Winnipeg, MB Canada; 5 Department of Allergy and Immunology, McGill University, Montreal, QC Canada; 6 Food Allergy Canada, Toronto, ON Canada; 7 Department of Pediatrics, Faculty of Medicine, University of British Columbia, Vancouver, BC Canada; 8 Department of Business and Administration, University of Winnipeg, Winnipeg, MB Canada; 9 Children’s Allergy and Asthma Education Centre, Winnipeg, MB Canada; 10 Institute of Environmental Medicine, Karolinska Institutet, Stockholm, Sweden

**Keywords:** Food allergy, Cost of illness, Financial burden, Intervention, Mixed-methods

## Abstract

**Background:**

Food allergy imposes considerable financial costs on families, but few programs are available in Canada to offset these costs. To fill this gap, we developed, piloted, and evaluated a program designed to address the financial burden of food allergy.

**Methods:**

The current study employed the use of an unblinded, crossover design. Participating families who began the study in the case condition received biweekly deliveries of food packages for 2 months, while those in the control condition received recipes and educational materials. Following the initial study period, the groups entered a one-month washout period and the conditions were reversed. During both conditions, an adult member of each participating family (“caregivers”) responded to a quantitative cost measure and completed a qualitative interview. Quantitative data were analysed using a series of linear mixed models. Qualitative data were analysed using thematic analysis.

**Results:**

A total of 14 participants were randomized to a sequence using Stata. However, 5 participants were dropped from the final quantitative sample due to a failure to complete one or more set of quantitative measures. Caregivers included in the final quantitative sample were 32.1 years old, on average, overwhelmingly female (89%), and had annual, after-tax, household income of $52,660.00 (SD=$23,188.92; CAD). Target children were largely under six years old (89%) and were evenly split between boys (44%) and girls (44%). Milk (67%), peanut (67%), and egg (67%) allergies were most common. Quantitative results revealed participants had non-significantly lower indirect costs in the food delivery condition ($724.56 vs. $797.83), largely because of lower food preparation costs ($561.41 vs. $656.15). In contrast, participants reported non-significantly higher direct costs when they were receiving the food packages ($678.47 vs. $655.56). Findings from the qualitative interviews suggest that this increase may reflect the fact that participants purchased more expensive grocery items in response to the cost savings afforded by the program.

**Conclusions:**

Participants derived several benefits from the program, but more research is needed to better understand how to maximize the impact of programs like NOURISH-US and to identify families most in need of financial support.

**Supplementary Information:**

The online version contains supplementary material available at 10.1186/s13223-025-00983-2.

## Background

Food allergy is a common pediatric condition, affecting approximately 7% of Canadian children [1]. While progress has been made towards developing therapies for the condition, they are neither curative, nor widely available, meaning that avoidance of the offending food is the primary management strategy for most individuals with the condition. The need for avoidance can result in considerable burden for children and their families given the integral role played by food in both physical nourishment and promoting social bonds [2–5]. Some of this burden is the impact on quality of life and manifests in anxiety surrounding the potential for accidental exposures and frustrations stemming from social impediments [2]. However, research suggests that pediatric food allergy also carries a considerable financial burden for families [6]. A substantial proportion of the financial impact of food allergy has been attributed to out-of-pocket costs associated with an allergen-friendly diet [6]. While estimates of these costs vary considerably across samples, Canadian research suggests that, prior to the pandemic, families with a child with a food allergy were spending approximately $190.00 (Canadian Dollars [CAD]) more per month on food relative to families with an age-matched, child without food allergy [7]. Following the outbreak of COVID-19, food spending among households managing food allergy, was found to increase by an additional $100-$200 dollars per month, depending on the family’s level of income [8]. While much of the direct cost of food allergy has been attributed to spending on food, research has found that families also incur out-of-pocket expenses related to the need for epinephrine auto-injectors and, in some cases, specialized childcare [6].

Beyond out-of-pocket costs, families raising a child with food allergy are also saddled with indirect costs, which are typically defined as the loss of time, productivity, or career opportunities [9]. Quantitative research has found that these excess costs partially stem from the fact that families with a child with food allergy spend more time interacting with the healthcare system because of their need for follow-up testing, prescription renewals, and, in some cases, emergency treatment of reactions [6]. Qualitatively, parents also describe devoting a considerable amount of time to label reading and the preparation of allergen-friendly foods [10–12].

Not surprisingly, American research suggests that the additional costs of managing a food allergy may leave some individuals at greater risk of food insecurity [13–15]. For instance, Treffeisen and colleagues, using a large, nationally-representative sample, found pediatric food allergy was associated with 39% increased odds of food insecurity relative to families without a child with food allergy [15]. While large population-based studies examining the risk of food insecurity among families managing food allergy have not been conducted in Canada, a smaller study by Harbottle and colleagues provides some evidence that food insecurity may also be elevated among households with a food-allergic child [16].

While individuals and families managing food allergy have been found to incur greater costs as a result of their condition, leaving them vulnerable to food insecurity, little financial support is currently available in Canada. To make matters worse, qualitative research suggests that households managing food allergy face challenges in their ability to fully utilize food banks due to concerns about cross contamination and a lack of allergen-friendly food [17, 18]. In light of this gap, we developed and piloted a dairy-allergy food supplement program, called NOURISH (i.e., patieNt-Oriented research to Understand and addRess Inequities of food accesS and insecurity amongst Households managing food allergy). Through this program, lower and middle-income families with a child under the age of six years with a dairy allergy were provided with biweekly deliveries of allergen-friendly food products over a six month period (For a full description see: [11, 19]). These deliveries were intended to not only reduce the financial costs of food allergy, but also the stress and anxiety associated with the financial burden. Consistent with our aims, participants qualitatively reported that the program helped to reduce both their food costs and stress, but quantitative differences in costs did not reach statistical significance [11, 19]. Participants also emphasized that the program could be improved by including a greater variety of products in the food packages [11].

Following completion of the project, we developed a revised version of the program, called NOURISH-US (i.e., patieNt-Oriented research to Understand and addRess Inequities of food accesS and insecurity amongst Households managing food allergy – a Unique intervention Study). Through this work, we attempted to address the limitations of NOURISH by not only broadening the variety of products included in each package, but by also widening the eligibility criteria to families who manage any food allergy in a child 12 years or younger. In light of these changes, the present study aimed to assess how the revised program affects the costs of families managing pediatric food allergy, using a mixed-methods, concurrent nested design.

## Methods

### Recruitment

Participants were recruited between April and September 2022 through a database maintained by the principal investigator, physical advertisements posted throughout the City of Winnipeg, Manitoba, Canada and through social media. This involved posting advertisements on the social media pages associated with the research team and those aimed at Winnipeg-based parenting and food allergy communities. Families were eligible to participate if their annual net family income after deducting all federal, and provincial taxes, was less than $70,000 CAD and they had a child 12 or under who had an Immunoglobulin E (IgE) or non-IgE-mediated food allergy. To ensure individuals met the eligibility criteria, participants were asked to provide a proof of allergy letter from their child’s allergist or pediatrician. Any costs incurred by families to obtain the letter were reimbursed with study funds. Our a priori recruitment target for the study was 40 participants. A sample size of 40 was deemed to be appropriate given that it would afford an 80% chance of detecting a $45.00 dollar difference in costs between the case and control periods at a two-sided alpha value of 0.05 and a standard deviation of $100.00 between the two conditions. All participants were enrolled by the first author.

### Intervention and design

**Fig. 1:**
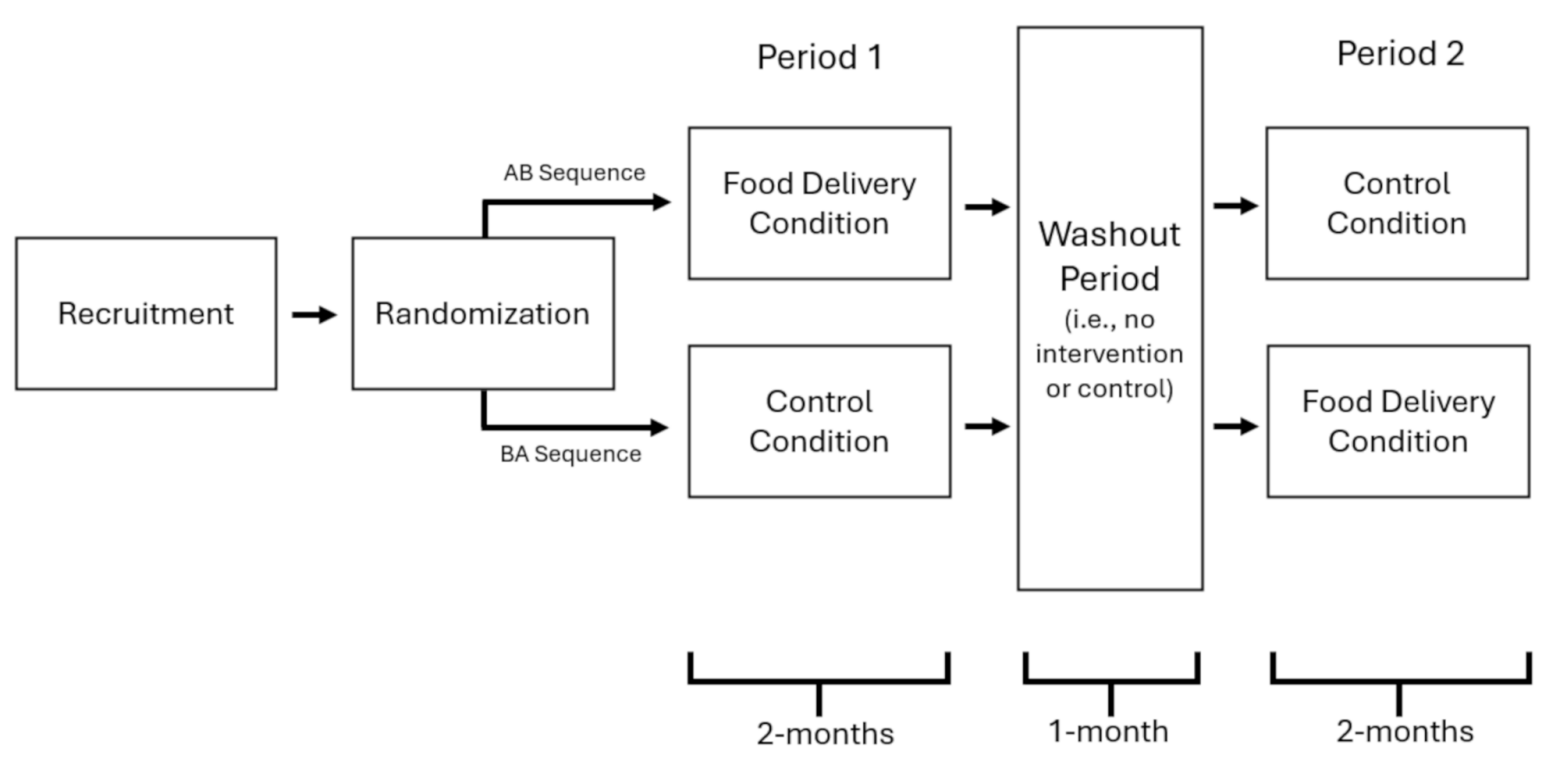
Overview of the AB/BA crossover design

The current study employed an AB/BA crossover design (See Fig. 1 for a visual representation). In this design, “AB/BA” is used to denote how the experimental conditions are ordered. Participants randomized to the AB sequence began the study in the case condition (i.e., A condition) and then moved to the control condition (i.e., B condition), while those in the BA sequence started the study in control condition (i.e., B condition) and then progressed to the case condition (i.e., A condition). This design was selected over parallel group designs for both ethical and statistical reasons. Because crossover designs compare the effect of the intervention within participants, they tend to be more powerful than parallel group designs. Moreover, they also afford all participants the opportunity to reap the potential benefits of the intervention because each individual participates in both the case and control condition.

The study’s design was informed by the CONSORT 2010 statement: extension to randomized crossover designs (please see Additional File 1 for a completed CONSORT checklist) [20]. Families who met the eligibility criteria and provided their informed consent were randomized to start the study in either the case (i.e., A) or control (i.e., B) condition. Randomization was performed by the first author, in Stata, using code disseminated by the World Bank [21]. In this approach, each participant is assigned a random number between 0 and 1, using the “runiform” function and then rank ordered based on their randomly assigned number. Participants whose random number placed them in the lower half of the sample were assigned to begin the study in the control condition, while those in the greater half were assigned to the case condition (hereafter referred to as the “food delivery condition”). This meant 50% of participants were assigned to begin the study in the food delivery condition and 50% were assigned to begin in the control condition. Given that the intervention consisted of biweekly deliveries of allergy-friendly foods, it was not feasible to blind the experimenters or the participants to the assigned sequences.

Participants randomized to begin the study in the food delivery condition received an allergy-friendly food basket every two-weeks for two months (i.e., period 1). Each basket was delivered to the participant’s home via contactless delivery by a pair of research assistants and contained allergen-friendly recipes, food products, and coupons for allergen-friendly food. Each basket was valued at approximately $75.00 CAD. Roughly 40%-50% of the basket’s total value was comprised of allergen-friendly food products and coupons donated by Daiya Foods Inc. These included allergen-friendly cheese products, convenience foods like macaroni and cheese, and coupons for free Daiya products. The other 50–60% of the basket’s value was largely devoted to ingredients that families could use to make the allergen-friendly recipe that was included in each package. If the value of the package was still under $75.00 after purchasing these ingredients, the remaining budget was spent on allergen-friendly staple items or snacks. Please see Additional File 2 for an example of the types of items included in the baskets.

Individuals who began the study in the control condition were provided with allergen-friendly recipes and food allergy-related management information every two weeks over the course of the first, 2-month study period. Following the initial period, both groups entered a 1-month washout period in which neither group received allergen-friendly foods, recipes, or other materials. This period was primarily aimed at ensuring families in the food delivery condition had ample opportunity to consume the food they were provided before crossing over into the control condition. At the end of the washout period, the experimental conditions were switched. This meant participants who received food packages and recipes in the first period began receiving educational materials and recipes; whereas, participants who began the study receiving educational materials and recipes received biweekly deliveries of food packages and recipes throughout the 2nd study period.

### Mixing of methods

The study employed a concurrent nested mixed-methods design. In this approach, the qualitative and quantitative data are collected concurrently, but one method is given priority [22]. Given that the current project was focused on financial costs, the quantitative data were primarily of interest, while the qualitative data were used to help explain the quantitative findings. The quantitative data were collected online through a series of questionnaires that participants completed about six weeks after the beginning of each condition. In addition to the quantitative measures, participants were also asked to complete a series of two phone interviews. One interview was completed near the end of the food delivery condition and the other near the end of the case condition. After the quantitative and qualitative data were collected and analysed, the findings from these two methods were integrated. The results from this integration are described in the discussion section of the current publication.

### Measures

Food allergy-related costs were a primary outcome of the current study and were measured using the Food Allergy Economic Questionnaire (FA-EcoQ), a validated, self-report measure designed to assess the direct and indirect costs associated with food allergy [23]. Direct costs describe out-of-pocket medical and non-medical expenses associated with the maintenance of one’s health [9]. Indirect costs, on the other hand, include costs stemming from lost time or productivity [9]. In the present study, direct costs included those incurred through the purchase of medication, groceries, and prepared meals (e.g., takeout, fast-food, and other restaurant meals). Indirect costs included lost time from work or school due to the child’s food allergy and time spent grocery shopping and preparing meals. Indirect costs were derived using a human capital-based method in which the number of hours lost were multiplied by the after-tax hourly wage reported by the family member incurring the cost [24]. If an individual was unemployed, their time was valued at the provincial after-tax minimum wage at the time of data analysis (i.e., $11.95 CAD). All reported costs reflect the previous month, unless stated otherwise.

As part of the FA-EcoQ, participants were also asked to complete several demographic questions related to the age, gender, education, and health history of each of their household’s members. The questionnaire also contained items related to employment and household income. In order to ease the burden on participants, several items that were irrelevant to the aims of the current study were omitted from the FA-EcoQ.

### Qualitative data collection

Qualitative data were collected from participating caregivers through a series of two phone interviews conducted approximately one week before the end of each study condition. Each interview was led by trained research assistants and made use of a semi-structured interview guide. The guides were tailored to the experimental condition that preceded the interview, but also contained some general questions on the costs of food allergy that were common to both interviews (Both guides can be found in Additional File 3).

### Data analyses

Descriptive analyses were used to characterize the composition of the sample. Afterwards, a series of linear mixed models were used to assess quantitative changes in costs between the food delivery and control conditions. Similar to multiple linear regression, linear mixed models can be used to assess the relationship between a continuous outcome variable and a series of predictors. However, unlike multiple regression, linear mixed models can handle repeated-measures data by modelling the correlation between repeated observations in the same participants (Interested readers can find a general equation that describes the conceptual basis of linear mixed models in Additional File 4). In each of the models used in the current study, condition (food delivery vs. control), period (fall vs. winter), and sequence (AB vs. BA) were specified as fixed effects, while participant was specified as a random effect to account for the non-independence between time-points. Given the small size of the sample, the Kenward-Rogers approximation was used to calculate the degrees of freedom for testing the fixed effects [25]. Stata 18 was used for each of these analyses (StataCorp; College Station, TX).

Qualitative data stemming from the interview transcripts were analysed by the second author through thematic analysis [26]. In this approach, the analyst organizes the data by applying short descriptions (i.e. codes) to relevant sections of text. In the current study, the analyst first read the transcripts to become familiar with their content. Following this initial reading, the transcripts were coded and afterwards the codes were reviewed for their inappropriateness. Inadequate codes were then revised, before a final coding of the transcripts was completed. Once coding was complete, the analyst looked for broader patterns among the codes, which are referred to as themes in the language of thematic analysis. Unlike codes, which typically express one idea, themes describe broad patterns in the data related to the research question and contain several different aspects organized around a central concept [26].

## Results

**Fig. 2:**
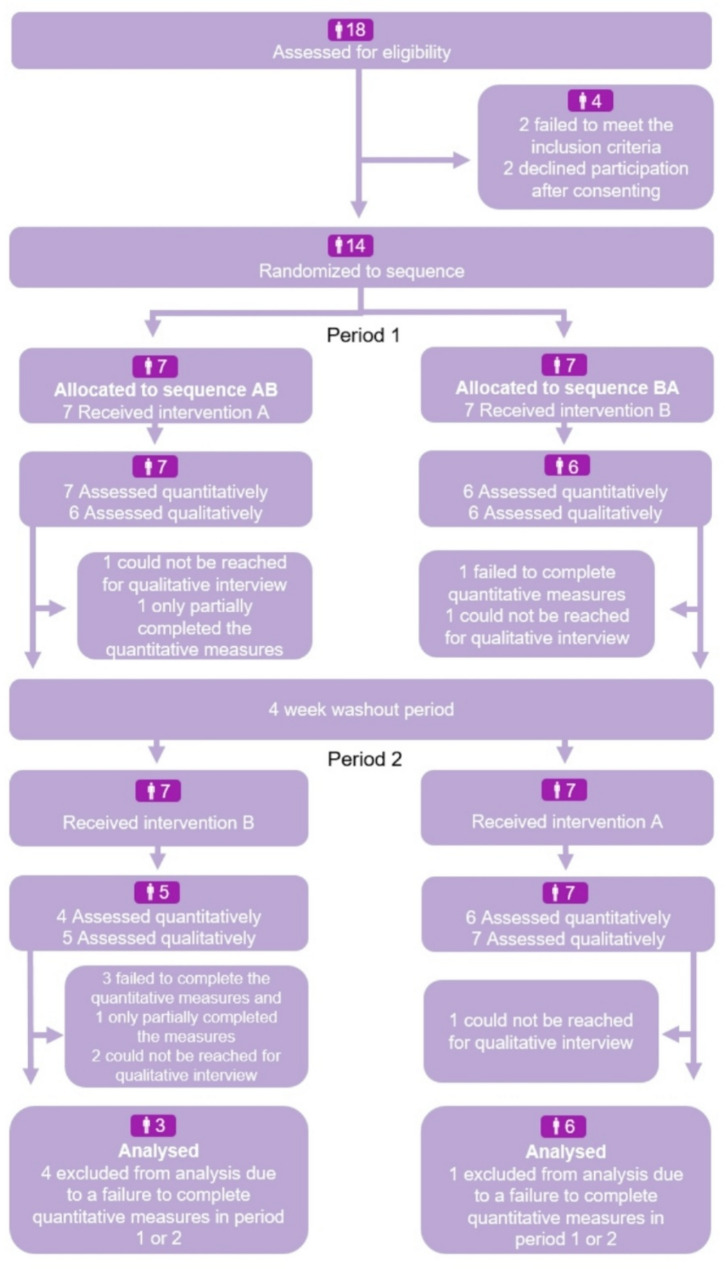
Flowchart depicting the progression of participants through the study procedures

In total, 14 participants consented to the study and were randomized to a sequence. However, only nine completed both sets of quantitative questionnaires (i.e., food delivery and control conditions). Because of the small size of the sample, we were not able to address the missing data through multiple imputation. For this reason, we used a completer only approach and limited the final quantitative sample to the nine families who completed both conditions (Please see Fig. 2 for a depiction of how participants progressed through the study’s phases). Relative to participants with complete quantitative data, participants with incomplete data were slightly younger, reported a lower level of education and income, and were less likely to have a spouse (Please see Additional File 5 for a comparison of the demographic attributes of families included in the final quantitative sample and those excluded as a result of missing data).

Of the families who were dropped from the final sample due to a failure to complete one or more sets of quantitative measures, four were from sequence AB and one was from sequence BA. As a result, the final allocation of participants varied across the two sequence groups (sequence AB = 3; sequence BA = 6). An investigation of the demographic characteristics of these groups revealed a greater proportion of participants in sequence AB had a university degree and were working at the time of the study. They also reported greater family incomes on average (Please see Table 1 for a summary of the demographic characteristics of each sequence group).

**Table 1:** Demographic and clinical characteristics

	Sequence AB (*n* = 3)			Sequence BA (*n* = 6)			Qualitative sample		
	%	n	Mean (*SD*)	%	n	Mean (*SD*)	%	n	Mean (*SD*)
Caregiver age		3	33.0 (6.6)		6	31.7 (4.4)		13	31.4 (4.8)
Caregiver gender									
Male	0%	0		17%	1		8%	1	
Female	100%	3		83%	5		92%	12	
Caregiver relationship status									
Spouse	33%	1		33%	2		46%	7	
No spouse	67%	2		67%	4		54%	6	
Caregiver education									
High school or less	0%	0		17%	1		31%	4	
Post-secondary degree, diploma, or certificate	100%	3		83%	5		69%	9	
Caregiver employment									
Employed	100%	3		33%	2		61%	8	
Unemployed, not seeking work	0%	0		0%	0		23%	3	
Unemployed, seeking work	0%	0		50%	3		7%	1	
Student	0%	0		17%	1		7%	1	
After-tax annual household income		3	$59,340.00 (11,024.83)		6	$49,320.00 (27,777.35)			$52,333.84 ($24,829.46)
Adjusted after-tax annual household income*			$31, 657.20 (7,606.77)			$26, 184.48 (15,037.79)			$26,637.20 (12,767.60)
Target child age		3	3.0 (2.6)		6	4.0 (2.8)			3.8 (2.6)
Target child gender									
Boy	33%	1		50%	3		54%	7	
Girl	67%	2		33%	2		38%	5	
Missing		0		17%	1		8%	1	
Target child food allergies									
Peanut	67%	2		67%	4		54%	7	
Milk	67%	2		67%	4		69%	9	
Egg	67%	2		67%	4		61%	8	
Tree nut	33%	1		33%	2		38%	5	
Shellfish	33%	1		17%	1		23%	3	
Fish	33%	1		17%	1		15%	2	
Sesame	33%	1		17%	1		15%	2	
Multiple food allergies									
Yes	67%	2		83%	5		85%	11	
No	33%	1		17%	1		15%	2	

Note. SD, standard deviation; *Income adjusted for household size by dividing the household’s total income by the square root of the number of members

As a whole, caregivers included in the final quantitative sample were approximately 32 years old on average (m = 32.1; SD = 4.8) and were overwhelmingly female (89%). They had an average after-tax annual household income of $52,660.00 (SD=$23,188.92; CAD) and most had some form of post-secondary education (89%). The majority of participants had only one child living in the home (56%), while the remainder cared for either two (22%) or three (22%). Target children were roughly evenly split between boys (44%) and girls (44%; 1 caregiver chose not to report their child’s gender) and were predominantly under the age of 6 years old (89%). Among these children, egg, milk, and peanut allergies were most common (67% each). The majority of the target children had more than one physician-diagnosed food allergy (78%). For most households included in the quantitative sample, the target child was the only member with food allergy (78%; See Table 1 for an overview of the sample’s demographics).

### Changes in costs

Findings from the current study revealed no statistically significant differences in direct costs between the food delivery and control conditions. While not statistically significant, monthly total direct costs were higher, on average, when participants were receiving the food packages (predictive marginal means [PMMs]: delivery condition=$678.47 per month; control condition=$655.56 per month; *b*=$22.90, *p* = 0.85). This effect was largely driven by greater spending on groceries (PMMs: delivery condition=$557.78 per month; control=$567.78 per month; *b*=$10.00, *p* = 0.91) and medication (PMMs: delivery condition=$29.51 per month; control condition=$17.86 per month; *b*=$11.65, *p* = 0.59). Participants also reported spending more on prepared meals when they were receiving the food deliveries, although the difference in costs between the two conditions was slight (PMMs: delivery condition=$81.18 per month; control condition=$79.93 per month; *b=*$1.25, *p* = 0.97; See Table 2 for a full overview of the quantitative findings).

Participants did report lower total indirect costs when they were receiving the food packages (PMMs: delivery condition=$724.56; control condition=$797.83; *b*= -$73.27, *p* = 0.56), but this difference failed to reach statistical significance. The lower indirect costs reported in the delivery condition, largely stemmed from a decrease in food preparation costs (PMMs: delivery condition=$561.41 per month; control condition=$656.15 per month; *b* = -$94.74, *p* = 0.38), although participants did also report lower monthly costs stemming from time lost from their work or school as a result of their child’s food allergy (PMMs: delivery condition=$12.31; control condition=$26.30; *b*=-$13.99, *p* = 0.19). Participants were found to have higher indirect costs stemming from time spent grocery shopping when they were receiving the food packages (PMMs: delivery condition=$150.84 per month; control condition=$115.38 per month; *b*=$35.46, *p* = 0.45), but again this effect was not statistically significant.

**Table 2:** A comparison of monthly direct and indirect costs between experimental conditions

	Control Condition	Case Condition					
	Marginal Mean*	Marginal Mean	n	Cohen’s f^2^	*b*	95%CI	*p*-value
*Total direct costs*	$655.56	$678.47	9	0.002	22.90	-248.94, 294.74	0.85
Total direct food costs	$637.71	$648.96	9	< 0.001	11.25	-243.19, 265.69	0.92
Grocery costs	$557.78	$567.78	9	0.001	10.00	-194.26, 214.29	0.91
Prepared meals	$79.93	$81.18	9	< 0.001	1.25	-75.42, 77.92	0.97
Medication costs	$17.86	$29.51	9	0.03	11.65	-37.56, 60.86	0.59
*Total indirect costs*	$797.83	$724.56	9	0.05	-73.27	-359.42, 212.87	0.56
Total indirect food costs	$771.53	$712.25	9	0.03	-59.28	-349.38, 230.81	0.64
Shopping costs	$115.38	$150.84	9	0.05	35.46	-70.38, 141.29	0.45
Food preparation costs	$656.15	$561.41	9	0.11	-94.74	-333.72, 144.24	0.38
Lost time from work/school^†^	$26.30	$12.31	9	0.30	-13.99	-36.76, 8.78	0.19

Note.*Predictive marginal means from a series of linear mixed models. ^†^refers to costs stemming from instances where the child’s caregivers are required to miss time from work or school because of the child’s food allergy.

b, unstandardized regression weight; 95%CI, 95% confidence interval

### Qualitative findings

A total of 13 participants completed the qualitative interview following the food delivery condition and 11 completed the control condition interview. Given that we were primarily interested in participants’ perceptions of the food packages, we included participants in the qualitative analyses even if they failed to complete the control condition interview. Participants in the qualitative sample were overwhelmingly female (92%) and tended to be in their early 30s (M = 31.4; SD = 4.8). Most reported having post-secondary education (77%). Among the target children, boys slightly outnumbered girls (boys = 54%; girls = 38%; prefer not to answer = 9%). The majority (85%) of these children were under six years old and had more than one food allergy (85%). Milk allergy was most common (69%), followed by allergies to egg (62%), and peanut (54%). For most households included in the qualitative sample, the target child was the only member with food allergy (71%).

Results from the qualitative interviews revealed one theme related to the impact of the NOURISH-US program on food costs. We called this theme: *Food subsidy eases household food costs and improves well-being.*

### Food subsidy eases household food costs and improves well-being

Throughout the interviews, participants described several benefits of the food supplement program, including direct cost savings. Naturally, because families were receiving biweekly deliveries of both specialized allergen-friendly food products and staple foods, they indicated that they no longer had to purchase certain items on their own, which they perceived as helping to reduce their grocery costs. For example, one participant remarked: *“I definitely think it [the program] has helped out because on the weeks that there is a delivery*,* I tend to wait for the delivery to see what type of food is delivered*,* so that I don’t buy the same thing. Yes I would say it has probably helped out like 30% of the cost maybe*,* like it has been substantial”.* Moreover, some families described how the provision of the meal kits not only reduced spending on groceries, but also the number of trips they were required to make to the store. Because of these savings, a number of families reported that they were empowered to spend more freely by saying “yes” to purchases they typically would avoid due to costs: *“I think it gave us more opportunity to buy other allergen friendly foods…I did look at things I would normally not buy cause they are kind of expensive*,* but I am like ok we have a little bit of extra money and now let’s try this or let’s just see. It gave us more opportunity to try other things for us.”*

Families also described how the program helped to reduce their stress and worry surrounding the introduction of new products and recipes. Because families were provided with products free of charge, caregivers described how they could try new products without having to worry that their child would dislike a potentially costly item: *“It has been nice for us because we have been able to try out new recipes and new products we did not know were out there.”*

Some participants also indicated that the provision of recipes helped reduce the time and effort they exerted finding new allergen-friendly meal ideas: “*The biggest thing for our family is trying to think of meals*.”. While the provision of recipes and their constituent ingredients was largely perceived as a benefit, some participants did express a desire to have a greater ability to customize the recipes and meal packages to their own liking and family size. That being said, the food subsidy program was described as a “*step in the right direction*” with some parents hoping it may one day expand to consider other household costs including those related to the purchase of epinephrine autoinjectors.

## Discussion

Findings from the current study paint an interesting, but somewhat mixed picture of the NOURISH-US program. Contrary to expectations, participants reported spending slightly more, on groceries, prepared meals, and medications when they were receiving the food packages, but despite this, many qualitatively reported deriving cost savings from the program. In contrast, participants were found to have lower total indirect food costs when they were in the food delivery condition and this effect was largely driven by decreased food preparation time. Interestingly, costs related to time spent food shopping were found to increase in the period when participants were provided with the food packages.

While the fact that direct costs were higher in the case condition certainly came as a surprise, the qualitative findings do appear to provide some insight into why participants reported spending more when they were receiving the food packages. During the qualitative interviews, several participants indicated that the biweekly deliveries of food provided them with the financial freedom to purchase grocery items that they would typically avoid due to cost. Naturally, this finding would seem to suggest that the slight increase in direct costs in the food delivery condition stems from the fact that the families were using the cost savings derived from the provision of food kits to purchase more expensive food items rather than allocate the money to savings or other goods and services. Although unexpected, this finding is consistent with both theory and research from behavioural economics, which has found that people tend to treat small windfalls, even when they are expected, as “found money” and are more likely to use them to purchase non-essential goods and services, rather than allocate the money to savings [27, 28].

Interestingly, while participants quantitatively reported higher direct costs when they were receiving the food packages, many reported deriving cost savings from the program during the qualitative interview. While it’s difficult to determine the cause of this discrepancy, it could be argued that participants were simply unaware that their purchases of more expensive grocery items in the food delivery condition increased their food costs over and above baseline. However, it may also be possible that references to cost savings made during the qualitative interviews referred not to overall spending, but to the fact that some of the items provided in the food packages replaced items they would normally be forced to pay for on their own.

By using the cost savings towards the purchase of novel grocery items, it is likely that participants were attempting to enhance their dietary variety, quality and/or convenience as research suggests that all three of these dietary components can be compromised through restriction diets [29, 30]. However, this practice may have had the unintended consequence of increasing indirect shopping costs in the food delivery condition as it likely requires more careful label reading to ensure the novel products are safe. That being said, it is unlikely that an increase in label reading fully accounts for the difference in indirect shopping costs between the two conditions given the magnitude of the effect.

Contrary to direct costs, participants did, interestingly, report fewer indirect food preparation costs when they were receiving the food packages, although this difference failed to reach statistical significance. Part of this effect may stem from the fact that the food packages contained, among other items, shelf-stable convenience foods (e.g., macaroni and cheese) and coupons for frozen meals and desserts. However, the allergen-friendly recipes contained within the packages may have also helped to reduce the indirect food preparation costs of families. A latent interpretation of this difference underscores the substantial time commitment dedicated to food preparation; indeed, our intervention, valued at $75 biweekly or about 23% of monthly food costs, contributed to a ~ 8% reduction in indirect costs reported by families managing food allergy.

During the interviews, participants described spending considerable time and effort finding meal ideas that met their family’s dietary needs. With this in mind, it is not surprising that participants reported deriving benefit from the provision of the allergen-friendly recipes as part of the program. While food allergy-related meal planning difficulties have not been described in the qualitative literature to date, these findings are consistent with previous quantitative work [31]. Arguably, more research is needed to better understand these issues; but, findings from the current study suggest that increasing access to allergen-friendly recipes may represent one low-cost strategy for reducing the burden of food allergy on families. For families in which children are involved in meal planning and food preparation, downstream benefits are diverse, ranging from more enjoyable mealtimes, to improved nutrition, and psychosocial and physical health [32, 33].

Naturally, the current study is subject to several limitations that may affect the interpretation of the findings. Perhaps most significantly, the study’s small sample size meant that it was underpowered to detect significant effects. It also means that the parameter estimates from the linear mixed models are likely to be less reliable and generalizable relative to those derived from a larger samples. Of course, the parameter estimates may have also been affected by differential participant attrition. Although the differences were not tested inferentially, participants who failed to complete the quantitative measures tended to report not having a spouse and lower levels of education and income. In light of these differences, it’s possible that estimates of the intervention’s effect are biased towards more highly educated, two-parent households.

The current study was also arguably limited by its use of a self-report measure of food allergy costs. Because participants were asked to retrospectively report on their food, medication, shopping, and meal preparation costs, it could be argued that their estimates may have been affected by recall bias. To limit the impact of bias, future investigations of similar programs should strive to employ more objective methods (e.g., collection of receipts) and methods that shorten the length of the recall period (e.g., daily diary-based methods and ecological momentary assessments).

It should also be mentioned that it is possible that participants may have continued to consume food provided in the food delivery condition while they were in the control condition, which may have attenuated the differences between the two conditions. It’s also possible that providing educational materials and recipes to participants in the control condition may have had some impact on the study’s outcomes by influencing the shopping habits and meal preparation behaviours of participants. Lastly, efforts aimed at expanding this intervention may be limited to larger urban centres given that it is unlikely to be cost effective in rural and remote communities where the food products would need to be shipped over large distances. With these limitations in mind, results from the current study should be interpreted with a degree of caution.

## Conclusion

Contrary to expectations, families did not report quantitative reductions in direct costs when they were receiving the food packages. Families were, however, found to derive several other benefits from the program, including lower food preparation costs, and greater dietary variety. While the program was described as a step in the right direction, more research is needed to better understand how to maximize the benefits provided by programs like NOURISH-US and to identify families most in need of financial support.

## Supplementary Information

Below is the link to the electronic supplementary material.

Additional File 1: CONSORT checklist for crossover designs

Additional File 2: Example food basket items

Additional File 3: Interview guides

Additional File 4: Conceptual linear mixed model equation

Additional File 5: Comparison of participants included in the final quantitative sample and those removed as a result of incomplete data

## Data Availability

Datasets generated and analyzed during the current study are not publicly available out of respect for participant privacy, but are available from the corresponding author upon reasonable request.

## Abbreviations

CAD: Canadian dollars
CIHR: Canadian Institutes of Health Research
FA-EcoQ: Food Allergy Economic Questionnaire
IgE: Immunoglobulin E
NOURISH: patieNt-Oriented research to Understand and addRess Inequities of food accesS and insecurity amongst Households managing food allergy
NOURISH-US: patieNt-Oriented research to Understand and addRess Inequities of food accesS and insecurity amongst Households managing food allergy – a Unique intervention Study
