# Supplementary material for: NOURISH-US: a mixed-methods, randomized crossover study of a program designed to reduce the financial burden of food allergy
Source: Allergy Asthma Clin Immunol. 2025 Aug 21;21:37. doi: 10.1186/s13223-025-00983-2 (PMC12369260; doi:10.1186/s13223-025-00983-2)
Supplement: Supplementary file 1 — Additional File 1: CONSORT checklist for crossover designs [file 13223_2025_983_MOESM1_ESM.docx]

| Additional File 1. CONSORT checklist for crossover designs | | | |
| --- | --- | --- | --- |
| Section/Topic | Item No | Description | Page No. |
| Title | 1a | Identification as a randomised crossover trial in the title | 1 |
| Abstract | 1b | Specify a crossover design and report all information outlined in table 2 | 3 |
| Introduction: |  |  |  |
| Background | 2a | Scientific background and explanation of the rationale | 5-7 |
| Objectives | 2b | Specific objectives or hypotheses | 7 |
| Methods: |  |  |  |
| Trial design | 3a | Rationale for a crossover design. Description of the design features including allocation ratio, especially the number and duration of periods, duration of washout period, and consideration of carry over effect | 8-9 |
| Change from protocol | 3b | Important changes to methods after trial commencement (such as eligibility criteria), with reasons | N/A |
| Participants | 4a | Eligibility criteria for participants | 8 |
| Settings and location | 4b | Settings and locations where the data were collected | 11-12, 13 |
| Interventions | 5 | The interventions with sufficient details to allow replication, including how and when they were actually administered | 9-11 |
| Outcomes | 6a | Completely defined prespecified primary and secondary outcome measures, including how and when they were assessed | 12-13 |
| Changes to outcomes | 6b | Any changes to trial outcomes after the trial commenced, with reasons | N/A |
| Sample size | 7a | How sample size was determined, accounting for within participant variability | 8 |
| Interim analyses and stopping guidelines | 7b | When applicable, explanation of any interim analyses and stopping guidelines | N/A |
| Randomisation: |  |  |  |
| Sequence generation | 8a | Method used to generate the random allocation sequence | 9 |
| Sequence generation | 8b | Type of randomisation; details of any restriction (such as blocking and block size) | 9 |
| Allocation concealment mechanism | 9 | Mechanism used to implement the random allocation sequence§ (such as sequentially numbered containers), describing any steps taken to conceal the sequence until interventions were assigned | N/A |
| Implementation | 10 | Who generated the random allocation sequence,§ who enrolled participants, and who assigned participants to the sequence of interventions | 9 |
| Blinding | 11a | If done, who was blinded after assignment to interventions (for example, participants, care providers, those assessing outcomes) and how | N/A |
| Similarity of interventions | 11b | If relevant, description of the similarity of interventions | N/A |
| Statistical methods | 12a | Statistical methods used to compare groups for primary and secondary outcomes which are appropriate for crossover design (that is, based on within participant comparison) | 13-14 |
| Additional analyses | 12b | Methods for additional analyses, such as subgroup analyses and adjusted analyses | 14 |
| Results |  |  |  |
| Participant flow (a diagram is strongly recommended) | 13a | The numbers of participants who were randomly assigned, received intended treatment, and were analysed for the primary outcome, separately for each sequence and period | 14-16 |
| Losses and exclusions | 13b | No of participants excluded at each stage, with reasons, separately for each sequence and period | 14-16 |
| Recruitment | 14a | Dates defining the periods of recruitment and follow-up | 8 |
| Trial end | 14b | Why the trial ended or was stopped |  |
| Baseline data | 15 | A table showing baseline demographic and clinical characteristics by sequence and period | Table 1 |
| Numbers analysed | 16 | Number of participants (denominator) included in the each analysis and whether the analysis was by original assigned groups | 14-16 |
| Outcomes and estimation | 17a | For each primary and secondary outcome, results including estimated effect size and its precision (such as 95% confidence interval) should be based on within participant comparisons. In addition, results for each intervention in each period are recommended | 18, 19, Table 2 |
| Binary outcomes | 17b | For binary outcomes, presentation of both absolute and relative effect sizes is recommended | N/A |
| Ancillary analyses | 18 | Results of any other analyses performed, including subgroup analyses and adjusted analyses, distinguishing prespecified from exploratory | 21-23 |
| Harms | 19 | Describe all important harms or untended effects in a way that accounts for the design (for specific guidance, see CONSORT for harms) | N/A |
| Discussion: |  |  |  |
| Limitations | 20 | Trial limitations, addressing sources of potential bias, imprecision, and if relevant, multiplicity of analyses. Consider potential carry over effects | 26-27 |
| Generalisability | 21 | Generalisability (external validity, applicability) of the trial findings | 26 |
| Interpretation | 22 | Interpretation consistent with results, balancing benefits and harms, and considering other relevant evidence | 23-25 |
| Other information: |  |  |  |
| Registration | 23 | Registration number and name of trial registry | N/A |
| Protocol | 24 | Where the full trial protocol can be accessed, if available | N/A |
| Funding | 25 | Sources of funding and other support (such as supply of drugs), role of funders | 1, 30 |
