# Supplementary material for: NOURISH-US: a mixed-methods, randomized crossover study of a program designed to reduce the financial burden of food allergy
Source: Allergy Asthma Clin Immunol. 2025 Aug 21;21:37. doi: 10.1186/s13223-025-00983-2 (PMC12369260; doi:10.1186/s13223-025-00983-2)
Supplement: Supplementary file 2 — Additional File 2: Example food basket items [file 13223_2025_983_MOESM2_ESM.docx]

Additional File 2 – Example Food Basket Items

| *Example food basket items* | | |
| --- | --- | --- |
| Item | Quantity | Approximate value |
| Recipe (see below) | 1 | - |
| Organic chicken bouillon cubes | 1 | $4.27 |
| 100% extra virgin olive oil | 1 | $6.94 |
| Carrot | 1 | $0.55 |
| Celery stalk | 1 | $5.33 |
| Sweet potato | 1 | $2.96 |
| Brown rice | 1 | $5.87 |
| Organic quinoa | 1 | $3.20 |
| Dried thyme leaves | 1 | $2.13 |
| White onion | 1 | $2.17 |
| Iodized table salt | 1 | $1.59 |
| Daiya free product coupon | 2 | $11.98 |
| Daiya Macaroni and Cheese | 2 | $12.58 |
| Shredded Daiya “cheese” | 1 | $6.49 |
| Block of Daiya “cheese” | 1 | $6.49 |
|  |  | Total = $72.55 |

Recipe: Quinoa with Brown Rice and Sweet Potatoes

Ingredients:

- Cooking spray
- 1 tablespoon extra virgin olive oil
- 1 medium onion chopped
- 1 medium carrot chopped
- 3 stalks of celery chopped
- 1 medium sweet potato peeled and chopped
- ¾ cup uncooked brown rice
- ¾ cup uncooked quinoa rinsed
- 3 cups wheat-free vegetable or chicken broth
- ½ teaspoon dried thyme leaves
- ½ teaspoon salt

Instructions:

1. Preheat the oven 350 [degrees] F. Spray an 8 quart casserole dish with cooking spray.

2. Heat the oil in a large skillet over medium heat. Add the onion, carrot, celery, and sweet potato to the skillet and sauté until tender.

3. Scoop the sautéed vegetables into the 8 quart casserole dish and add the quinoa, brown rice, broth, thyme, and salt. Stir to combine.

4. Bake in a preheated oven for 30-40 minutes or until the liquid has been absorbed and the grains are tender. Serve immediately.
