# Supplementary material for: NOURISH-US: a mixed-methods, randomized crossover study of a program designed to reduce the financial burden of food allergy
Source: Allergy Asthma Clin Immunol. 2025 Aug 21;21:37. doi: 10.1186/s13223-025-00983-2 (PMC12369260; doi:10.1186/s13223-025-00983-2)
Supplement: Supplementary file 3 — Additional File 3: Interview guides [file 13223_2025_983_MOESM3_ESM.docx]

Additional File 3. *Interview guides*

Control condition interview guide

**Introductory questions^[[1]](#footnote-1)^**

1. Would you please tell me a bit about yourself and your family?
2. What made you think that your child had a food allergy? How old was he/she?
3. What did you know about food allergy before your child was diagnosed?
4. How is your family doing during the COVID-19 pandemic?
   1. Work changes
   2. School/child care changes
   3. Physically overall
   4. Emotionally overall

**Content questions (with probing questions)**

1. What kinds of changes have you had to make because of the food allergy?^[[2]](#footnote-2)^
2. Please share a bit about what it has been like to buy allergen-friendly food with the increase in grocery prices we’ve seen over the past year. How do the costs compare to prior to the pandemic?
3. How does your family perceive the costs of allergen-friendly foods? // What does your family think about the costs of these foods?
   1. Any excess costs (e.g., Does your family spend more because of your child’s food allergy?)
      1. What products are more expensive?
   2. How well can your family absorb these costs?
   3. Changes in purchasing of other household items because of the costs
   4. Does your family purchase products with precautionary allergy labelling (e.g., may contain labels)?
      1. If yes, was this decision motivated by costs?
4. As you know, you’re currently in the control phase of the study where you’re just receiving educational materials. What do you think of the educational materials you’ve received so far?
   1. Have they been helpful? Why or why not?
   2. Were they easy to understand?
   3. Are there any food allergy topics you’d like to know more about?
      1. For instance, advocacy, management, science, therapies, prevention, etc.
5. In the new year you’ll start to receive biweekly deliveries of allergen-friendly food. Do you think an allergen-friendly food delivery program, like this, will benefit your family?
   1. If yes, how so?
   2. If no, why not?
6. Are food deliveries the “right” way to help families, or are there other approaches that would better suit families? Please explain.
   1. For instance, food packages vs. tax credits vs. benefit cheques
   2. If food packages are preferable, is the delivery system preferable to pick-up?

**Closing questions**

1. We've talked a lot today about what it is like to have a child with a food allergy. Is there anything that I should have asked, but did not?
2. Would you like to add anything else?
3. Now, I will provide a brief summary about what we talked about. Please correct me if anything is incorrect. (provide summary) How was that? Did I capture what you said or did I make some mistakes. (correct if necessary).
4. Now that you've heard the summary, is there anything else that you would like to add?

Case condition interview guide

**Introductory questions^[[3]](#footnote-3)^**

1. Would you please tell me a bit about yourself and your family?
2. What made you think that your child had a food allergy? How old was he/she?
3. What did you know about food allergy before your child was diagnosed?
4. How is your family doing during the COVID-19 pandemic?
   1. Work changes
   2. School/child care changes
   3. Physically overall
   4. Emotionally overall

**Content questions (with probing questions)**

1. What kinds of changes have you had to make because of the food allergy?^[[4]](#footnote-4)^
2. Please share a bit about what it has been like to buy allergen-friendly food with the increase in grocery prices we’ve seen over the past year. How do the costs compare to prior to the pandemic?
3. How does your family perceive the costs of allergen-friendly foods? // What does your family think about the costs of these foods?
   1. Any excess costs (e.g., Does your family spend more because of your child’s food allergy?)
      1. What products are more expensive?
   2. How well can your family absorb these costs?
   3. Changes in purchasing of other household items because of the costs
   4. Does your family purchase products with precautionary allergy labelling (e.g., may contain labels)?
      1. If yes, was this decision motivated by costs?
4. As you know, you’re participating in a study of an allergy-friendly food program. What do you think of the program so far?
   1. What do you like?
      1. Frequency of delivery
      2. Product confidence, taste
      3. Did the program have any impact on your food costs, as far as you can tell?
         1. If yes, how so? Has it affected your spending habits (purchasing more food, more expensive food items, spending more on other products/services, saving more?)
         2. If no, why?
      4. Alleviate concerns re food costs, food insecurity
      5. Improved mood, less stress, less worry about costs/access/cross-contamination
   2. Were there limitations or drawbacks of the program?
      1. For instance, lack of choice, difficulties incorporating foods in diet, undesirable products, etc.
      2. What could be improved?
5. Going forward, in what capacity would you like this program to continue.
6. What would you tell policy makers about this program?
7. Are food deliveries the “right” way to help families, or are there other approaches that would better suit families? Please explain.
   1. For instance, food packages vs. tax credits vs. benefit cheques
   2. If food packages are preferable, is the delivery system preferable to pick-up?

**Closing questions**

1. We've talked a lot today about what it is like to have a child with a food allergy. Is there anything that I should have asked, but did not?
2. Would you like to add anything else?
3. Now, I will provide a brief summary about what we talked about. Please correct me if anything is incorrect. (provide summary) How was that? Did I capture what you said or did I make some mistakes. (correct if necessary).
4. Now that you've heard the summary, is there anything else that you would like to add?

1. Only asked in period 1. In period 2, participants were just asked to remind the interview who was living in their household, who had allergies, and what they were allergic to. [↑](#footnote-ref-1)
2. Only asked in period 1. [↑](#footnote-ref-2)
3. Only asked in period 1. In period 2, participants were just asked to remind the interview who was living in their household, who had allergies, and what they were allergic to. [↑](#footnote-ref-3)
4. Only asked in period 2. [↑](#footnote-ref-4)
