# Supplementary material for: NOURISH-US: a mixed-methods, randomized crossover study of a program designed to reduce the financial burden of food allergy
Source: Allergy Asthma Clin Immunol. 2025 Aug 21;21:37. doi: 10.1186/s13223-025-00983-2 (PMC12369260; doi:10.1186/s13223-025-00983-2)
Supplement: Supplementary file 4 — Additional File 4: Conceptual linear mixed model equation [file 13223_2025_983_MOESM4_ESM.docx]

Additional File 4 – Conceptual linear mixed model equation

y = *β*_0_+Σ*β*_1_X_1_ + *γ* + *ε*

Where:

y = the outcome variable

β0 = the global intercept

*β*_1_ = fixed effect coefficients

X_1_ = fixed effect variables

*γ* = random effect variance

ε = residual variance
