# Supplementary material for: NOURISH-US: a mixed-methods, randomized crossover study of a program designed to reduce the financial burden of food allergy
Source: Allergy Asthma Clin Immunol. 2025 Aug 21;21:37. doi: 10.1186/s13223-025-00983-2 (PMC12369260; doi:10.1186/s13223-025-00983-2)
Supplement: Supplementary file 5 — Additional File 5: Comparison of participants included in the final quantitative sample and those removed as a result of incomplete data [file 13223_2025_983_MOESM5_ESM.docx]

| Additional File 5. *Comparison of participants included in the final quantitative sample and those removed as a result of incomplete data* | | | | | | | |
| --- | --- | --- | --- | --- | --- | --- | --- |
|  | Complete Quantitative Data | | | | Incomplete Quantitative Data | | |
|  | % | n | Mean (*SD*) | % | | n | Mean (*SD*) |
| Caregiver age |  | 9 | 32.1 (4.8) |  | | 4 | 29.8 (5.12) |
| Caregiver gender |  |  |  |  | |  |  |
| Male | 11% | 1 |  | 0% | | 0 |  |
| Female | 89% | 8 |  | 100% | | 4 |  |
| Caregiver relationship status |  |  |  |  | |  |  |
| Spouse | 67% | 6 |  | 25% | | 1 |  |
| No spouse | 33% | 3 |  | 75% | | 3 |  |
| Caregiver education |  |  |  |  | |  |  |
| High school or less | 11% | 1 |  | 75% | | 3 |  |
| Post-secondary degree, diploma, or certificate | 89% | 8 |  | 25% | | 1 |  |
| After-tax annual household income |  |  | $52,660.00 ($23,188.92) |  | |  | $48,600  ($24,829.46) |
| Target child age |  |  | 3.40 (2.66) |  | |  | 3.5 (2.38) |
| Target child sex |  |  |  |  | |  |  |
| Male | 44% | 4 |  | 75% | | 3 |  |
| Female | 44% | 4 |  | 25% | | 1 |  |
| Missing | 11% | 1 |  | 0% | | 0 |  |
| Target child food allergies |  |  |  |  | |  |  |
| Peanuts | 67% | 6 |  | 25% | | 1 |  |
| Milk | 67% | 6 |  | 75% | | 3 |  |
| Eggs | 67% | 6 |  | 50% | | 2 |  |
| Tree nuts | 22% | 2 |  | 50% | | 2 |  |
| Shellfish | 22% | 2 |  | 25% | | 1 |  |
| Fish | 22% | 2 |  | 0% | | 0 |  |
| Sesame | 22% | 2 |  | 0% | | 0 |  |
| Multiple food allergies |  |  |  |  | |  |  |
| Yes | 78% | 7 |  | 100% | | 4 |  |
| No | 22% | 2 |  | 0% | | 0 |  |
